# Prevalence of underweight, overweight and obesity among Palestinian school-age children and the associated risk factors: a cross sectional study

**DOI:** 10.1186/s12887-019-1842-7

**Published:** 2019-12-09

**Authors:** Saad Al-Lahham, Nidal Jaradat, Mohammad Altamimi, Ola Anabtawi, Alma Irshid, Malik AlQub, Majdi Dwikat, Fouad Nafaa, Lama Badran, Rawan Mohareb, Reema Haji, Tareq Aqqad, Sadeq Jayyab, Budour Abu Ghosh, Rina Taher, Hamzeh Al Zabadi

**Affiliations:** 1 Department of Biomedical Sciences, Faculty of Medicine and Health Sciences, An-Najah National University, P.O. Box 7, Nablus, Palestine; 2 Department of Pharmacy, Faculty of Medicine and Health Sciences, An-Najah National University, Nablus, Palestine; 3 Department of Nutrition and food technology, Faculty of Agriculture and Veterinary Medicine, An-Najah National University, Nablus, Palestine; 4 Department of Surgery, Rafidia Hospital, Nablus, Palestine; 5 Public Health Department, Faculty of Medicine and Health Sciences, An-Najah National University, Nablus, Palestine

**Keywords:** Childhood obesity, Childhood overweight, Childhood underweight, Dietary factors and socio-demographic factors

## Abstract

**Background:**

Childhood obesity is rising in developed and developing countries, while childhood underweight is rising mainly in developing countries. Childhood underweight has been shown to increase a child’s risk of rapid weight gain. Overweight and obese children are more likely to become obese adults, which increases the risk of type-II diabetes and cardiovascular diseases. Studies concerning obesity among Palestinian children are scarce. The prevalence of obesity among Palestinian children has increased from 3 to 6% within 5 years in comparison to the worldwide rise from 1 to 7%, within 41 years. We aim to determine the current prevalence of underweight, overweight and obesity among Palestinian school-age children and to assess the role of dietary and sociodemographic factors.

**Methodology:**

A cross sectional study was conducted in Palestine in 2017. A total of 1320 school-age children and their 2640 corresponding parents were recruited. A written questionnaire was filled out by the parents. Anthropometric indices were measured and categorized according to the Center for Disease Control and prevention (CDC).

**Results:**

The mean ± SD age of the children was 9.5 ± 1.5 years and 48.8% were females. The prevalence of underweight, overweight and obesity among the children was approximately 7.3% (95% CI = 5.9–8.8%), 14.5% (95% CI = 12.7–16.6%) and 15.7% (95% CI = 13.8–17.8%) respectively. Multinomial logistic regression analysis demonstrated a significant correlation of waist circumference, age, gender and living place with the body mass indexes of the students.

**Conclusion:**

Our findings highlighted the accelerated increase in the prevalence of underweight, overweight and obesity (37.5%) among Palestinian children within a very short time in comparison to the globe. Therefore, Interventions aiming to prevent obesity and underweight at an early stage might be vital to avoid obesity later in life and its health-related co-morbidities, e.g. type-II diabetes and cardiovascular diseases.

## Background

Obesity is escalating at an alarming rate worldwide, affecting children and adults in both developed and developing countries. According to the World Health Organization report in 2016, more than 1.9 billion adults (~ 39%) were overweight. Of these, over 600 million were obese (~ 13%) worldwide [1]. However, the prevalence of obesity alone among men and women of the USA population was 37.9 and 41.1% respectively [2]. The trends in obesity prevalence among adults in Palestine are similar to those of the USA, 30% among men and 49% among women [3, 4]. Concerning childhood obesity, in 2016, the prevalence of obesity worldwide and in the USA has been shown to be approximately 7% [1] and 18.5% respectively [2, 5, 6]. Studies concerning obesity among Palestinian children are scarce.

Obesity presents a major risk to health. It has been shown to be responsible for an estimated 216,000 deaths accounting for about 1 in 10 deaths in US adults [7]. Obesity increases the risk of chronic diseases such as metabolic syndrome, insulin resistance syndrome, cardiovascular diseases, type-II diabetes mellitus and some cancers [8]. According to the Palestinian Ministry of Health, these diseases are the leading causes of death in Palestine, accounting for approximately 50% of deaths [9]. Furthermore, it has been estimated that obesity-related illnesses in adults cost $209.7 billion of the US national medical care costs, which equals 20.6% of the US national health expenditures [10]. On the other hand, underweight among children and adolescents is associated with higher risk of infectious diseases and leads to overweight and obesity in adulthood [11]. Consequently, obesity in Palestine requires serious attention due to the high prevalence in adults and scarcity of studies on children, who constitute approximately 50% of the Palestinian population [12].

There are a few earlier Palestinian studies concerning childhood obesity and underweight which focused on the prevalence while very little attention was paid to the risk factors. Furthermore, earlier studies were either self-reported or had a small range of students’ ages and most of them concerned with older ages (> 12 years old). These studies are old and the last one was conducted in 2009, in which it has been shown that the prevalence of overweight and obesity among Palestinian school age children was approximately 13 and 6% respectively [13, 14]. Therefore, this is the first time (1) to determine the prevalence of underweight, overweight and obesity amongst Palestinian children with younger and greater age diversity (between 6 and 12 years old) and (2) to assess the role of some environmental factors such as dietary factors, physical activities, and socio-economic and demographic factors.

## Methods

### Study design, sample size and population

This is a cross-sectional study, which was conducted in the governorate of Nablus, in the north of Palestine, between June 2017 and December 2017. The total population of Nablus was 387,240, which represented 8.2% of the total population of the West Bank (PCBS-2017). The total number of students studying in both private and governmental schools in Nablus was 65,169 students (2016–2017). In order to obtain a representative sample, the schools enrolled were selected from various areas of the governorate and were either governmental or private schools. Included students were from the city, a village and refugee camps. However, the students who reside in the refugee camps, included in this study, were those studying in private or governmental schools, not in schools belonging to the United Nations Relief and Works Agency for Palestine Refugees (UNRWA). Unfortunately, we were not able to obtain permission from UNRWA to include its students.

The inclusion criteria were; age between 6 and 12 years old, acceptance to participation, ability to give the anthropometric records and enrollment in governmental or private schools. The proportion of students enrolled from private schools represented 14% of the sample. This is due to the limited number of private schools and students in this sector in Palestine as the governmental (public) sector is the main provider of education and health in Palestine.

### Data collection procedure

Permission was obtained from the department of school’s health, ministry of education, Palestine. This study was approved by An-Najah National University Institutional Review Board (8/August/2016). Then the researchers visited the chosen schools to inform them about the survey and to distribute the consent forms. After having received the consents from the parents/guardians of children, the researchers recorded the height, weight and waist circumference (WC) for each student in the examination room of the school. The researchers also recorded age as well as gender of each student. Then the students were asked to take the questionnaire to their homes to be filled out by one of their parents or guardians. The questionnaire was designed to gather personal, socioeconomic and demographic and life-style information (Food frequency, physical activity, transportation and electronic device use). The questions included in the questionnaire were tailored to the objectives of the study and the population sample. This is based on risk factors associated with childhood obesity investigated in many earlier studies worldwide [15, 16]. However, only the factors applied to the context of Nablus governorate were included in our questionnaire. Regarding the food frequency questionnaire, unfortunately, there is no Palestinian standardized and validated food frequency questionnaire for this specific dietary assessment and this age group (6–12 years). Therefore, the nutritionists in the research team conducted a 24-h recall for a small group of children (6–12 years) to assess the food items consumed by this group of children in the governorate of Nablus. The identified food items were included in the modified FFQ based on the one validated by Hamdan et al. [17].

### Anthropometric measurements

Weight was measured to the nearest 0.1 kg in light clothing and bare feet using a digital balance (Omron, BF511). Standing height was measured to the nearest 0.5 cm using a portable tape measure without shoes. WC was recorded using an inelastic flexible standard plastic measuring tape on minimal clothing in the abdominal area in a standing position and measurement was recorded to the nearest 0.1 cm. All measurements were taken twice using a standard method of anthropometric assessment described earlier [18] and then averages were used to calculate body mass index (BMI). All children were fasting when measurements took place.

BMI was calculated as weight in kilograms divided by height in meters squared. It was categorized based on age and sex-specific cut-off values of the 2000 Centers for Disease Control and Prevention (CDC) growth charts. The categories were underweight (< 5th percentile), normal weight (5th to 85th percentile), overweight (85th to 95th percentile), and obese (> 95th percentile) [19].

### Statistical analysis

Descriptive statistics and bivariate analysis were performed to assess the relationship between the independent variables (sociodemographic and questionnaire responses) and the child BMI as a dependent variable. BMI was calculated by dividing the child weight in kg by the child square height in meter. Therefore, the child weight and/or height were never considered as independent variables in any stage of the analysis due to strong correlation with the dependant variable (BMI).

Child’s BMI was categorized as underweight, normal, overweight and obese according to 2000 CDC reference. Chi square test was used to assess the relationship between categorical independent variables and dependent variable, while ANOVA was used to assess the relationship between the continuous independent variables and dependent variable. A *P* value less than 0.05 was always considered significant. Variables showed significance in the bivariate analysis was then entered in the multinomial logistic regression analysis using enter method.

## Results

### Participants’ characteristics

The total number of students aged (6–12) years old in private and governmental sectors in the province was 65,169 students. To achieve an acceptable margin of error up to 5% at the 95% confidence level, the sample size should be at least 400 participants. We expected a low response rate due to psychological aspects of obesity among the Palestinian people; therefore, we chose a convenient sample of 1320 to ensure obtaining a good study response rate. Clearly, this 1320 sample size gave a margin of error of 3%. It should be noted that this study estimated different categories of BMI (i.e., normal, underweight, overweight and obesity), therefore, calculating the sample size based on any single BMI category would not be accurate. We distributed consent forms to 1320 families and got 100% agreement to participate in this study. We recorded anthropometric indices, age and gender for their children. Then children were asked to take the questionnaire to their homes to be filled by one of their parents/guardians. In this study phase, the response rate was approximately 58% (i,e., not all families filled the requested information). The mean ± SD of the age was 9.5 ± 1.5 years and 48.8% were females. As shown in Table 1, approximately 58.9% of the children were living in the city, 34.5% in villages and 6.6% in refugee camps. The majority (73%) of parents declared that their children walked to school, while 5.8, 25.5 and 68.8% of the children have spent less than 30, 60 and above 60 min in daily physical activities. On the other hand, 15.4, 37.4 and 47.3% of children have spent less than 30, 60 and above 60 min respectively watching on a screen as declared by their parents. The majority of the mothers (83.6%) had at least high school education, while it was 76.9% in the case of the fathers. However, only 20% of the mothers were employed. Fifth of the mothers (21%) had a caesarean section. Mothers declared that feeding regimens in the first 6 months of their children’s lives were 68.3, 7.7% or 23.9% for breastfeeding, formula milk or a combination of both respectively. Regarding parents’ anthropometrics, 38.5% of mothers and 46.1% of fathers were overweight, while 18.3 and 27.2% of mothers and fathers were obese, respectively. Almost one third (29.8%) of the mothers had gestational diabetes and only 4% of the mothers were smokers. Children had various dietary habits which were described in Table 2.

**Table 1:** Association between socio-demographic and physical activity factors with child’s BMI

		BMI of Children					
Characteristics		Normal n (%)	Overweight n (%)	Obese n (%)	Underweight n (%)	Total n (%)	*p*-value
Living place	City	229 69.6%	42 12.8%	50 15.2%	8 2.4%	329 100.0%	0.002
	Village	115 59.6%	33 17.1%	29 15.0%	16 8.3%	193 100.0%	
	Refugees Camp	19 51.4%	5 13.5%	12 32.4%	1 2.7%	37 100.0%	
Maternal education level	Not educated	8 80.0%	2 20.0%	0 .0%	0 .0%	10 100.0%	0.928
	Primary school	52 65.8%	10 12.7%	15 19.0%	2 2.5%	79 100.0%	
	High school	149 64.5%	37 16.0%	35 15.2%	10 4.3%	231 100.0%	
	College	35 64.8%	6 11.1%	10 18.5%	3 5.6%	54 100.0%	
	BA or more	105 62.5%	24 14.3%	30 17.9%	9 5.4%	168 100.0%	
Paternal education level	Not educated	7 63.6%	3 27.3%	1 9.1%	0 .0%	11 100.0%	0.315
	Primary school	74 64.9%	19 16.7%	15 13.2%	6 5.3%	114 100.0%	
	High school	144 64.0%	35 15.6%	34 15.1%	12 5.3%	225 100.0%	
	College	34 56.7%	12 20.0%	13 21.7%	1 1.7%	60 100.0%	
	BA or more	91 68.9%	10 7.6%	26 19.7%	5 3.8%	132 100.0%	
Maternal work	Housewife	279 64.9%	60 14.0%	71 16.5%	20 4.7%	430 100.0%	0.882
	Others	68 63.0%	18 16.7%	18 16.7%	4 3.7%	108 100.0%	
House hold income (NIS)*	< 2000	72 69.2%	11 10.6%	16 15.4%	5 4.8%	104 100.0%	0.717
	2000–2999	110 65.5%	28 16.7%	23 13.7%	7 4.2%	168 100.0%	
	3000–3999	90 60.8%	21 14.2%	32 21.6%	5 3.4%	148 100.0%	
	> 4000	69 62.7%	16 14.5%	19 17.3%	6 5.5%	110 100.0%	
Maternal BMI	Normal	149 70.0%	34 16.0%	22 10.3%	8 3.8%	213 100.0%	0.049
	Overweight	118 60.2%	25 12.8%	45 23.0%	8 4.1%	196 100.0%	
	Obese	54 58.1%	17 18.3%	20 21.5%	2 2.2%	93 100.0%	
	Underweight	4 57.1%	1 14.3%	1 14.3%	1 14.3%	7 100.0%	
Paternal BMI	Normal	98 75.4%	15 11.5%	12 9.2%	5 3.8%	130 100.0%	0.005
	Overweight	152 65.5%	37 15.9%	33 14.2%	10 4.3%	232 100.0%	
	Obese	74 54.0%	20 14.6%	38 27.7%	5 3.6%	137 100.0%	
	Underweight	4 100.0%	0 .0%	0 .0%	0 .0%	4 100.0%	
Having gestational diabetes	Yes	155 68.5%	31 13.7%	31 13.7%	10 4.4%	227 100.0%	0.773
	No	347 65.0%	76 14.2%	88 16.5%	23 4.3%	534 100.0%	
Mode of delivery	Normal	291 66.3%	60 13.7%	67 15.3%	21 4.8%	439 100.0%	0.199
	Caesarean	69 59.0%	20 17.1%	25 21.4%	3 2.6%	117 100.0%	
Feeding	Breastfeeding	246 66.0%	51 13.7%	59 15.8%	17 4.6%	373 100.0%	0.341
	Formulas	28 66.7%	3 7.1%	8 19.0%	3 7.1%	42 100.0%	
	Both	79 60.3%	25 19.1%	24 18.3%	3 2.3%	131 100.0%	
Smoking during pregnancy	Yes	10 66.7%	2 13.3%	3 20.0%	0 .0%	15 100.0%	0.740
	No	202 56.4%	60 16.8%	75 20.9%	21 5.9%	358 100.0%	
Transporting means to school	Family Car or School Bus	89 59.3%	20 13.3%	36 24.0%	5 3.3%	150 100.0%	0.031
	Walking	274 67.5%	59 14.5%	55 13.5%	18 4.4%	406 100.0%	
Screen time (minutes)	< 30	52 61.9%	14 16.7%	12 14.3%	6 7.1%	84 100.0%	0.069
	60	145 71.1%	28 13.7%	24 11.8%	7 3.4%	204 100.0%	
	> 60	154 59.7%	37 14.3%	56 21.7%	11 4.3%	258 100.0%	
Physical activity (minutes)	< 30	18 56.3%	7 21.9%	5 15.6%	2 6.3%	32 100.0%	0.560
	60	86 61.4%	18 12.9%	30 21.4%	6 4.3%	140 100.0%	
	> 60	249 66.2%	54 14.4%	57 15.2%	16 4.3%	376 100.0%	
WC mean + SD (N)		Walking	68.2 ± 6.5 (106)	78.1 ± 9.0 (119)	57.1 ± 4.8 (33)	756	0.000
Birth weight (Kg) mean + SD (N)		3.2 ± 0.6 (349)	3.3 ± 0.7 (80)	3.2 ± 0.6 (91)	2.9 ± 0.5 (24)	544	0.154
Child’s daily allowance* mean + SD (N)		2.7 ± 1.4 (356)	2.9 ± 1.3 (80)	3.0 ± 1.4 (92)	2.8 ± 1.9 (24)	552	0.557
Total number of family members mean + SD (N)		6.4 ± 2.2 (356)	6.4 ± 1.7 (80)	6.2 ± 1.5 (92)	6.8 ± 1.3 (24)	552	0.592

*:NIS: New Israeli Shekel

**Table 2:** Association between dietary factors and child’s BMI

		BMI of Children					
Characteristics		Normal n (%)	Overweight n (%)	Obese n (%)	Underweight n (%)	Total n (%)	p-value
Having breakfast	Never	25 67.6%	5 13.5%	4 10.8%	3 8.1%	37 100.0%	0.239
	Once a month	28 59.6%	7 14.9%	7 14.9%	5 10.6%	47 100.0%	
	Twice a week	43 64.2%	11 16.4%	11 16.4%	2 3.0%	67 100.0%	
	Most days	190 61.9%	45 14.7%	59 19.2%	13 4.2%	307 100.0%	
	More than once a day	48 78.7%	7 11.5%	6 9.8%	0 .0%	61 100.0%	
Meat intake	Never	15 60.0%	6 24.0%	3 12.0%	1 4.0%	25 100.0%	0.139
	Once a month	26 55.3%	11 23.4%	5 10.6%	5 10.6%	47 100.0%	
	Twice a week	153 63.8%	32 13.3%	48 20.0%	7 2.9%	240 100.0%	
	Most days	141 68.8%	25 12.2%	29 14.1%	10 4.9%	205 100.0%	
	More than once a day	14 63.6%	5 22.7%	2 9.1%	1 4.5%	22 100.0%	
Fish intake	Never	63 60.0%	19 18.1%	20 19.0%	3 2.9%	105 100.0%	0.076
	Once a month	208 67.1%	44 14.2%	45 14.5%	13 4.2%	310 100.0%	
	Twice a week	50 59.5%	8 9.5%	21 25.0%	5 6.0%	84 100.0%	
	Most days	14 66.7%	2 9.5%	4 19.0%	1 4.8%	21 100.0%	
	More than once a day	4 57.1%	1 14.3%	0 .0%	2 28.6%	7 100.0%	
Diary intake	Never	7 77.8%	1 11.1%	1 11.1%	0 .0%	9 100.0%	0.497
	Once a month	19 67.9%	6 21.4%	3 10.7%	0 .0%	28 100.0%	
	Twice a week	55 62.5%	13 14.8%	15 17.0%	5 5.7%	88 100.0%	
	Most days	164 65.1%	42 16.7%	38 15.1%	8 3.2%	252 100.0%	
	More than once a day	108 64.3%	16 9.5%	34 20.2%	10 6.0%	168 100.0%	
Rice and Pasta intake	Never	3 42.9%	3 42.9%	0 .0%	1 14.3%	7 100.0%	0.094
	Once a month	27 79.4%	5 14.7%	1 2.9%	1 2.9%	34 100.0%	
	Twice a week	100 60.2%	25 15.1%	31 18.7%	10 6.0%	166 100.0%	
	Most days	182 66.7%	36 13.2%	48 17.6%	7 2.6%	273 100.0%	
	More than once a day	40 59.7%	11 16.4%	11 16.4%	5 7.5%	67 100.0%	
Cereals at breakfast	Never	126 61.2%	34 16.5%	34 16.5%	12 5.8%	206 100.0%	0.511
	Once a month	76 72.4%	10 9.5%	17 16.2%	2 1.9%	105 100.0%	
	Twice a week	78 63.9%	19 15.6%	21 17.2%	4 3.3%	122 100.0%	
	Most days	49 60.5%	15 18.5%	12 14.8%	5 6.2%	81 100.0%	
	More than once a day	9 56.3%	2 12.5%	5 31.3%	0 .0%	16 100.0%	
Bread intake	Never	3 60.0%	1 20.0%	0 .0%	1 20.0%	5 100.0%	0.4
	Once a month	23 65.7%	5 14.3%	5 14.3%	2 5.7%	35 100.0%	
	Twice a week	22 57.9%	7 18.4%	9 23.7%	0 .0%	38 100.0%	
	Most days	118 67.8%	26 14.9%	24 13.8%	6 3.4%	174 100.0%	
	More than once a day	185 64.2%	39 13.2%	51 17.7%	14 4.9%	289 100.0%	
Fruits intake	Never	4 66.7%	0 .0%	2 33.3%	0 .0%	6 100.0%	.895
	Once a month	21 67.7%	4 12.9%	4 12.9%	2 6.5%	31 100.0%	
	Twice a week	72 63.2%	16 14.0%	22 19.3%	4 3.5%	114 100.0%	
	Most days	146 65.2%	30 13.4%	39 17.4%	9 4.0%	224 100.0%	
	More than once a day	107 63.3%	30 17.8%	23 13.6%	9 5.3%	169 100.0%	
Fruit juice intake	Never	18 47.4%	9 23.7%	9 23.7%	2 5.3%	38 100.0%	0.45
	Once a month	49 62.8%	14 17.9%	13 16.7%	2 2.6%	78 100.0%	
	Twice a week	116 61.7%	26 13.8%	37 19.7%	9 4.8%	188 100.0%	
	Most days	128 69.9%	21 11.5%	25 13.7%	9 4.9%	183 100.0%	
	More than once a day	40 69.0%	9 15.5%	7 12.1%	2 3.4%	58 100.0%	
Vegetables intake	Never	6 42.9%	3 21.4%	3 21.4%	2 14.3%	14 100.0%	0.695
	Once a month	34 63.0%	8 14.8%	9 16.7%	3 5.6%	54 100.0%	
	Twice a week	90 65.7%	20 14.6%	21 15.3%	6 4.4%	137 100.0%	
	Most days	157 66.5%	34 14.4%	39 16.5%	6 2.5%	236 100.0%	
	More than once a day	63 61.8%	13 12.7%	19 18.6%	7 6.9%	102 100.0%	
Salad intake	Never	13 54.2%	3 12.5%	5 20.8%	3 12.5%	24 100.0%	0.515
	Once a month	34 68.0%	9 18.0%	5 10.0%	2 4.0%	50 100.0%	
	Twice a week	161 64.9%	39 15.7%	40 16.1%	8 3.2%	248 100.0%	
	Most days	111 64.2%	19 11.0%	33 19.1%	10 5.8%	173 100.0%	
	More than once a day	32 64.0%	9 18.0%	8 16.0%	1 2.0%	50 100.0%	
Chips intake	Never	11 52.4%	1 4.8%	6 28.6%	3 14.3%	21 100.0%	0.337
	Once a month	35 72.9%	7 14.6%	4 8.3%	2 4.2%	48 100.0%	
	Twice a week	69 66.3%	16 15.4%	17 16.3%	2 1.9%	104 100.0%	
	Most days	129 63.2%	31 15.2%	33 16.2%	11 5.4%	204 100.0%	
	More than once a day	109 64.5%	24 14.2%	30 17.8%	6 3.6%	169 100.0%	
Chocolate intake	Never	10 52.6%	6 31.6%	2 10.5%	1 5.3%	19 100.0%	0.016
	Once a month	28 63.6%	5 11.4%	8 18.2%	3 6.8%	44 100.0%	
	Twice a week	87 61.7%	28 19.9%	24 17.0%	2 1.4%	141 100.0%	
	Most days	132 60.6%	27 12.4%	43 19.7%	16 7.3%	218 100.0%	
	More than once a day	91 75.2%	14 11.6%	14 11.6%	2 1.7%	121 100.0%	
Carbonated Beverages intake	Never	72 60.5%	18 15.1%	22 18.5%	7 5.9%	119 100.0%	0.581
	Once a month	81 69.8%	13 11.2%	14 12.1%	8 6.9%	116 100.0%	
	Twice a week	114 63.0%	28 15.5%	33 18.2%	6 3.3%	181 100.0%	
	Most days	58 66.7%	16 18.4%	12 13.8%	1 1.1%	87 100.0%	
	More than once a day	27 64.3%	5 11.9%	8 19.0%	2 4.8%	42 100.0%	
Energy drinks intake	Never	305 64.2%	71 14.9%	78 16.4%	21 4.4%	475 100.0%	0.749
	Once a month	12 60.0%	3 15.0%	4 20.0%	1 5.0%	20 100.0%	
	Twice a week	20 71.4%	0 .0%	7 25.0%	1 3.6%	28 100.0%	
	Most days	8 72.7%	2 18.2%	1 9.1%	0 .0%	11 100.0%	
	More than once a day	5 55.6%	2 22.2%	1 11.1%	1 11.1%	9 100.0%	
Diet carbonated drinks intake	Never	312 65.4%	69 14.5%	75 15.7%	21 4.4%	477 100.0%	0.087
	Once a month	14 53.8%	6 23.1%	5 19.2%	1 3.8%	26 100.0%	
	Twice a week	13 68.4%	1 5.3%	4 21.1%	1 5.3%	19 100.0%	
	Most days	6 46.2%	1 7.7%	6 46.2%	0 .0%	13 100.0%	
	More than once a day	3 42.9%	3 42.9%	0 .0%	1 14.3%	7 100.0%	
Sweets (Knafeh, Cakes)	Never	23 62.2%	8 21.6%	4 10.8%	2 5.4%	37 100.0%	0.04
	Once a month	101 63.1%	20 12.5%	33 20.6%	6 3.8%	160 100.0%	
	Twice a week	147 62.6%	40 17.0%	42 17.9%	6 2.6%	235 100.0%	
	Most days	66 73.3%	7 7.8%	9 10.0%	8 8.9%	90 100.0%	
	More than once a day	15 65.2%	5 21.7%	1 4.3%	2 8.7%	23 100.0%	
Fast food intake	Never	26 57.8%	8 17.8%	9 20.0%	2 4.4%	45 100.0%	0.656
	Once a month	119 66.1%	24 13.3%	32 17.8%	5 2.8%	180 100.0%	
	Twice a week	137 62.3%	31 14.1%	38 17.3%	14 6.4%	220 100.0%	
	Most days	55 71.4%	11 14.3%	8 10.4%	3 3.9%	77 100.0%	
	More than once a day	16 61.5%	6 23.1%	4 15.4%	0 .0%	26 100.0%	
Nuts intake	Never	23 59.0%	7 17.9%	9 23.1%	0 .0%	39 100.0%	0.829
	Once a month	118 65.2%	25 13.8%	29 16.0%	9 5.0%	181 100.0%	
	Twice a week	131 62.1%	37 17.5%	33 15.6%	10 4.7%	211 100.0%	
	Most days	62 69.7%	8 9.0%	15 16.9%	4 4.5%	89 100.0%	
	More than once a day	16 66.7%	3 12.5%	4 16.7%	1 4.2%	24 100.0%	

### Prevalence of underweight, overweight and obesity and the role of gender and age

**Fig. 1:**
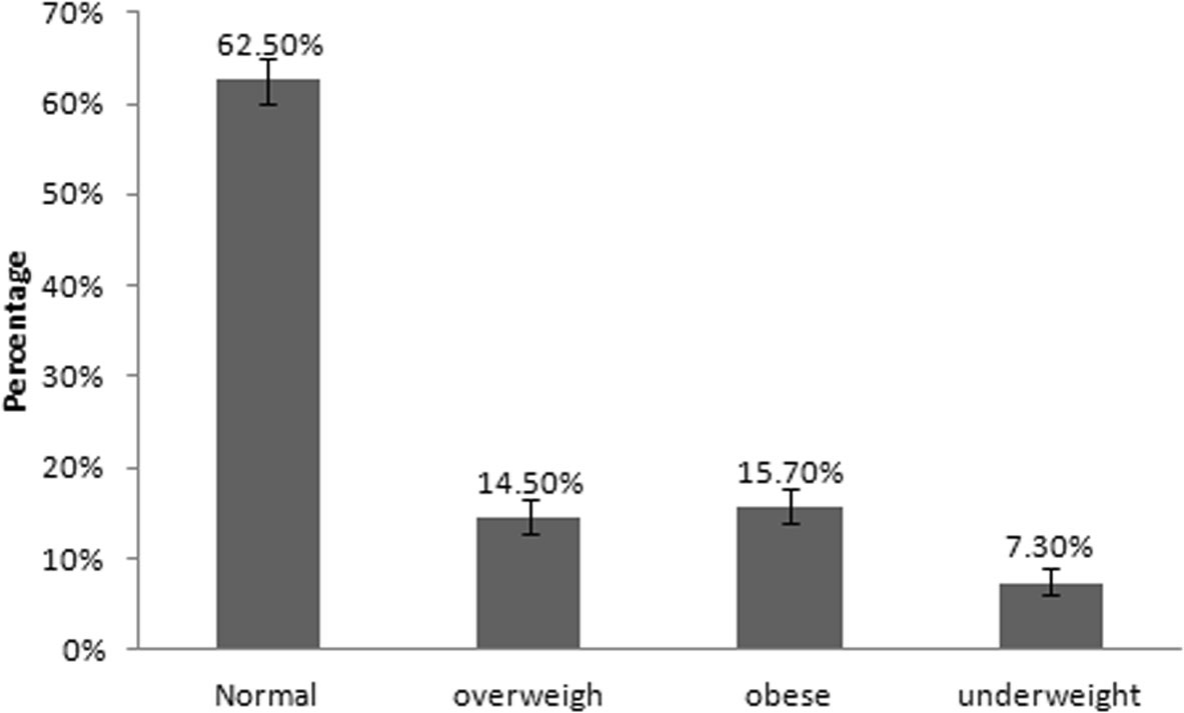
Prevalence of underweight, overweight and obesity among school age children. Error bars represent CI. *N* = 1320

**Fig. 2:**
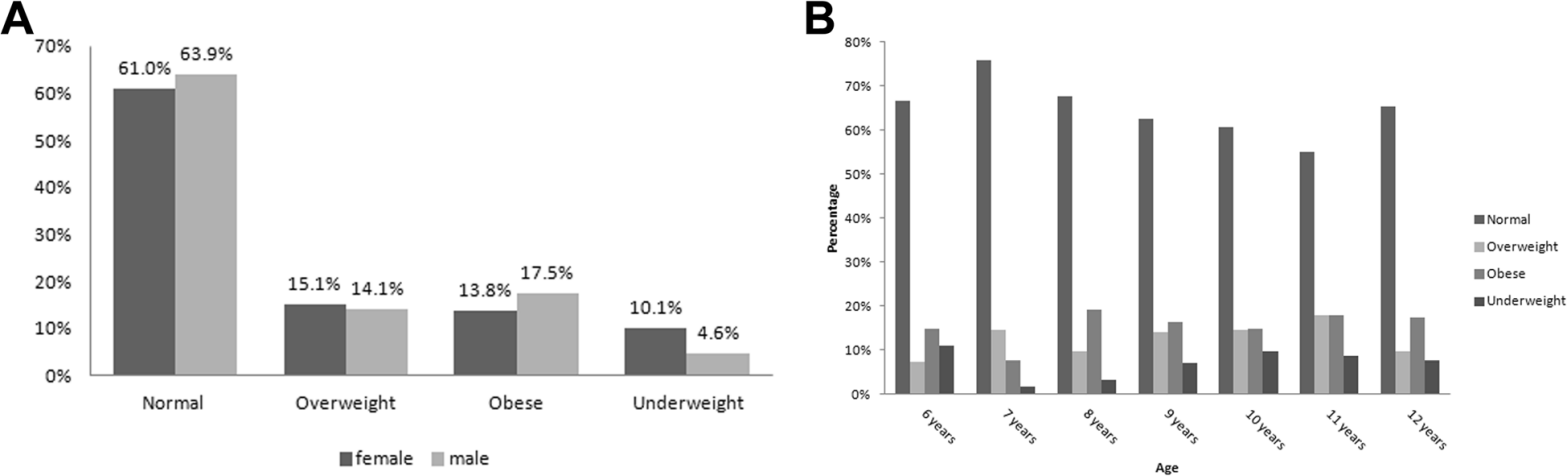
**a** Distribution of Gender among BMIs categories: **b** Distribution of age among BMIs categories. *N* = 1320

As shown in Fig. 1, the prevalence of underweight, overweight and obesity among the 1320 students were approximately 7.3% (95% CI = 5.9–8.8%), 14.5% (95% CI = 12.7–16.6%) and 15.7% (95% CI = 13.8–17.8%) respectively. As shown in Fig. 2 a and b, we have conducted a chi-square analysis for gender (male, female) and age (6,7,8,9,10,11 and 12 years) among BMI categories (normal, underweight, overweight and obesity) and the chi-square *p*-value in both cases was found to be < 0.001. The percentage of the normal-weight female was 61% while the percentage of the overweight female was 15.1%. On the other hand, the percentage of those of 9 years old and had normal weight was 65%, while only 5% of the same age was underweight. Also, those who were 12-year-old and normal weight represented nearly 66%, while nearly 17% were obese for the same age. For the distribution of the percentages of gender and age among BMI categories refer to Fig. 2 a and b.

### Bivariate analysis

Out of 1320 questionnaires sent to the families of the children, only 761 were filled out. Such questionnaires were used to calculate the association of students’ BMIs with sociodemographic, physical activity and dietary factors. As described in Table 1 (sociodemographic factors), living place (*P* = 0.001), maternal BMI (*P* = 0.049), paternal BMI (*P* = 0.005) and WC (*P* < 0.00049) were shown to have significant associations with BMIs of school-age children. However, parental education, maternal work, household income, gestational diabetes, mode of delivery, breastfeeding, smoking, birth weight, daily allowance and total number of family members had no associations with BMI categories. Levels of physical activity are shown in Table 2. It was found that only transportation means had a significant effect (*P* = 0.031). Screen time had no significant effect, however, it has a borderline effect (*P* = 0.069). The food frequency questionnaire (Table 2) has revealed that chocolate (*P* = 0.016) and sweets (*P* = 0.04) had significant effect on the BMIs of the students.

### Multinomial logistic regression analysis

To further analyze and understand the relationship between the aforementioned factors, variables showed to be significant in the above bivariate analysis were entered in multinomial logistic regression analysis where we built a multivariate model to assess the multi-factorial effect of independent variables on child’s BMI. This model showed that WC, age, gender and living place remained significantly associated with BMIs of the students (Table 3). Interestingly, this model indicated that the variables in the model could explain 72% of the variation in BMIs of the students. Females were more likely to be underweight (OR = 23.5; 95% CI = 3.87–141.77). Students living in the city were more likely to be obese (OR = 3.6; 95% CI = 1.09–11.81), simultaneously, they were less likely to be underweight (OR = 0.04; 95% CI = 0.01–0.21). Older ages were significantly less likely to be overweight and obese (ORs and 95%CIs were; 0.67 and 0.51–0.88 for overweight and; 0.39 and 0.26–0.58 for obese). Students with higher WC were significantly more likely to be overweight and obese (ORs and 95%CIs were; 1.4 and 1.27–1.50 for overweight and; 1.8 and 1.59–2.02 for obese). However, higher WC were significantly less likely to be underweight (OR and 95% CI were 0.72 and 0.60–0.87).

**Table 3:** Multinomial regression analysis model for the variables associated with child’s BMI ^**#**^

Variable	Overweight			Obese			Underweight			Pseudo R-Square (Nagelkerke)
	B	SE	OR (95%CI)	B	SE	OR (95%CI)	B	SE	OR (95%CI)	
Gender* Female	0.47	0.35	1.6 (0.8–3.20)	0.87	0.54	2.24 (0.78–6.44)	3.16	0.92	23.47 (3.87–141.77)	0.72
Screen time‡ < 30 min	0.81	0.48	2.25 (.89–5.70)	0.8	0.73	2.22(0.54–9.25)	0.05	0.8	1.05 (0.22–5.04)	
60 min	−0.24	0.39	0.79 (0.37–1.69)	− 0.94	0.58	0.39 (0.13–1.20)	− 0.36	0.64	0.70 (0.20–2.42)	
Fish intake± Never	−0.40	0.45	0.67 (0.28–1.60)	−0.6	0.63	0.55 (0.16–1.87)	− 0.12	0.76	0.89 (0.20–3.92)	
Chocolate± Never	0.75	0.83	2.11 (0.41–10.77)	0.05	1.42	1.05 (0.06–16.99)	2.68	1.39	14.61 (0.96–221.74)	
Diet beverage± Never	−0.50	0.53	0.61 (0.22–1.70)	− 0.74	0.76	0.48 (0.11–2.13)	1.18	1.17	3.25 (0.33–32.14)	
Sweets intake± Never	0.52	0.68	1.68 (0.44–6.34)	−0.66	0.98	0.52 (0.08–3.49)	0.70	0.96	2.01 (0.31–13.18)	
Living place© City	0.41	0.41	1.50 (0.68–3.34)	1.28	0.61	3.58 (1.09–11.81)	−3.11	0.79	0.04 (0.01–0.21)	
Transporting means to school° Bus	0.21	0.39	1.23 (0.58–2.65)	0.6	0.53	1.82 (0.64–5.15)	−1.03	0.76	0.36 (0.08–1.57)	
Mothers weight^	0.001	0.02	1.00 (0.97–1.03)	0.02	.02	1.02 (0.97–1.06)	0.02	0.03	1.02 (0.97–1.08)	
Fathers BMI^	−0.002	.04	1.00 (0.92–1.08)	0.09	0.06	1.09 (0.98–1.22)	0.10	0.06	1.11 (0.98–1.25)	
Age^	−0.40	0.14	0.67 (0.51–0.88)	−0.95	0.21	0.39 (0.26–0.58)	0.07	0.23	1.07 (0.68–1.68)	
WC^	0.32	0.04	1.38 (1.27–1.50)	0.58	0.06	1.79 (1.59–2.02)	−0.33	0.1	0.72 (0.60–0.87)	

#: Reference category (Normal weight); *: male; ‡:> 60 min; ±: yes;©: village; °: walking; ^: OR: odd ratio. CI: confidence intervals

## Discussion

Overweight and obese children are more likely to become obese adolescents and adults, with major short and long-term health and economic consequences [20–22]. Obesity-related disorders, such as metabolic syndrome, insulin resistance, type 2 diabetes and cardiovascular diseases, which are known to occur only in adults now appear in children [23]. Strikingly, childhood underweight increases the risk of overweight and obesity later in life [11]. Therefore, determining the current prevalence and understanding the factors related to obesity and/or underweight in children are vital.

In the current study, we found that the prevalence of overweight and obesity were 14.5 and 15.7% respectively. This is similar to the prevalence in the USA and some neighboring Middle Eastern countries such as Jordan [24]. Nevertheless, it is still lower than the prevalence of other Middle Eastern countries such as Saudi Arabia (20–28%). The observed prevalence of obesity in this study, but not overweight, is higher than the prevalence determined in earlier studies in Palestine. In 2004, the prevalence of overweight and obesity among Palestinian children [25] was approximately 13.3 and 3.2% respectively; while in 2009, it was approximately 13 and 6% respectively [13, 26]. Together with the current findings, a fast rise in the prevalence of obesity, but not overweight, in Palestinian children has been revealed within a very short period of time, which is alarming. The prevalence of obesity worldwide has risen from 1 to 7% within 41 years [1]; similarly, it has increased in Palestine, however, in a very accelerated manner from 3.2 to 15.7% within 14 years. This agrees with the prevalence of Middle Eastern countries, where Palestine geographically is located [27].

Although studies concerning childhood obesity are scarce in Palestine; the attention paid to underweight prevalence and its associated factors, is even less. In the present study, we have found that the prevalence of underweight is approximately 7.3%, which was 2 folds higher than the prevalence observed in an earlier Palestinian study of a similar age [13] and of that in the USA [28]. The prevalence of underweight found in the current study, was even higher than the prevalence in similar age group of neighbouring countries such as Jordan (5.7%) [24]. Our data suggest that the prevalence of underweight in Palestinian children has doubled within a short period of time (10 years).

This is the first time in Palestine to study the association of BMI of young children (6–12 years) with a wide range of factors, such as sociodemographic, physical activity and dietary factors. Employing bivariate analysis, we found that gender, age, living place, maternal and paternal BMI, WC, transportation means and chocolate and sweet intake were significantly associated with BMIs of children. However, employing the multivariate model, we found that gender, age, living place and WC remained significant, while the rest failed to show such significant associations. This could be due to the notion that all students who had one or more missing data were removed from the multivariate model. However, this missing data was random with no specific pattern of characteristics among participants. For example, no missing data was shown regarding gender, age and WC (i.e., these variables were entered in the multivariate model).

Our results demonstrate the coexistence of underweight, overweigh and obese children in Palestine at the same time, which contribute to approximately 37.5% of unhealthy body weight, which was found to be almost 2 folds higher than described earlier (2009) in Palestine [13]. This is a common and a major problem in low and middle income countries and is described as a double burden of malnutrition [29, 30]. Our data agree with Massad, et al. [13] findings, where that underweight was associated with gender and physical activity of the Palestinian children. In contrast to our study, Massad, et al. [13] showed an association between underweight and unemployed mothers. Our results also agree with Mikki’s, et al. [14] findings, who reported that living place had a significant effect on BMI, while education of mothers and family size had no association. On the other hand, they did not show an association with age, in contrast to our findings.

Although the questionnaires were self reported, the prevalence of many variables was similar to the ones described in earlier Palestinian studies, suggesting that our data is reliable. For example the prevalence of caesarean section observed in our study (21%) corresponds with a recent study in Palestine [31] and with the global rate [32]. We also found that 56.5% of mothers and 73.4% of fathers were overweight and obese, which agrees with earlier Palestinian studies [2, 33–35]. The percentage of the smoking mothers (4%) is similar to the percentage described earlier (3.5%) [36].

We found that approximately 30% of the mothers had gestational diabetes mellitus (GDM). To our knowledge, this is the first study in Palestine to investigate the prevalence of GDM. The observed prevalence is extremely high in comparison to neighboring countries such as Jordan (13.5–17.3% [37, 38] and Saudi Arabia (24.2%) [39] and European countries (2–6%) [40]. This high prevalence could be an overestimate as a result of self-reporting; however, this indicates the need to further study the prevalence of GDM and its related risk factors in Palestine in the future.

We found some factors that were significantly associated with BMIs, using bivariate analysis, but not by multivariate analysis. This could be due to the limited size of the sample of these factors. For example, the majority of parents were overweight and obese and 4% of the sample was smoking mothers. This is one of the limitations of the current study, which make it difficult to investigate its relation to BMIs of the children. Another limitation was self reported questionnaires. In addition, this study was limited to Nablus governorate; however, together with earlier and recent studies in Palestine, obesity prevalence is high and increasing in children.

## Conclusion

Prevalence of underweight, overweight and obesity among Palestinian school age children was found to be high. It has been rising dramatically in an accelerated manner within a short period of time in comparison with the international figures. Rise in obesity prevalence is most probably due to the fast urbanization and the transition from conventional to western-life style after the establishment of the Palestinian authority. Furthermore, unfortunately, Palestine is located in a conflict area that is politically and economically unstable, where people are exposed to food insecurity and movement restrictions at the time of conflicts. This may unfortunately enable the coexistence of underweight, overweigh and obese children in Palestine at the same time, which result in approximately 40% of children at increased health risks due to being in an unhealthy weight. In our study, we found that gender, age, living place and WC have been associated with BMIs of the Palestinian children. As a consequence, our findings call for a serious attention to obesity in Palestinian children, who constitute approximately 50% of the total population in Palestine [12]. Therefore, obesity prevention should be a national public health priority to reduce and prevent it at an early stage, and consequently preventing its related disorders and economic consequences.

## Data Availability

The datasets used and/or analysed during the current study are available from the corresponding author on reasonable request.

## Abbreviations

BMI: Body mass index
CDC: Center for Disease Control and prevention
GDM: Gestational diabetes mellitus
NIS: New Israeli Shekel
UNRWA: United Nations Relief and Works Agency for Palestine Refugees
WC: Waist circumference
